# Response to: Comment on “Evaluation of Antiviral Therapy Performed after Curative Therapy in Patients with HBV-Related Hepatocellular Carcinoma: An Updated Meta-Analysis”

**DOI:** 10.1155/2016/5210902

**Published:** 2016-08-16

**Authors:** Peng Yuan, Yeben Qian, Peng Chen

**Affiliations:** Department of Hepatobiliary Surgery, The First Affiliated Hospital of Anhui Medical University, Hefei 230032, China

Thanks are due to you for this letter and we would like to respond regarding some questions raised about our meta-analysis.

In this letter, the authors questioned the index used in the meta-analysis and recommended hazard ratio (HR) instead of relative risk (RR) [1]. HR is truly a good index for survival analysis of one disease because it considers the effects of time and it worked when extracting data from studies we selected; however, RR is appropriate for comparing the control group and treatment group (NAs group) here, as it has equal effects to OR.

The total number of patients was the same only in 1-year and 3-year recurrence, but, in OS and DFS, the numbers were different [2].

Disease-free survival has been defined as no recurrence of HCC in our article [2].

Randomized trials were really what we wanted to study in our meta-analysis; however, due to a lack of enough studies and data, this was unfortunately not possible [2].

We are not sure if postoperative NAs therapy has no impact on patients' short-term survival, for the reason that HBV reactivation usually occurs one month after operations were performed [3], so that is why we performed this meta-analysis. And as our results show, we do not agree with the comment that NAs have no short-term effects on survival and recurrence [1].

We would like to thank again the authors for this valuable letter and also for their concerns. We would like to receive further suggestions on our study.
